# Western European hedgehog’s *(Erinaceus europaeus)* head arteries

**DOI:** 10.1007/s11259-024-10568-w

**Published:** 2024-11-19

**Authors:** Jakub Jędrzej Ruszkowski, Maciej Zdun, Marcin Bartłomiej Arciszewski

**Affiliations:** 1 Department of Animal Anatomy, Poznan University of Life Sciences, Wojska Polskiego 71C, Poznan, 60-625 Poland; 2 Department of Animal Anatomy and Histology, University of Life Sciences in Lublin, Akademicka 12, Lublin, 20-950 Poland

**Keywords:** Anatomy, Angiology, Cephalic, Insectivore

## Abstract

The Western European Hedgehog *(Erinaceus europaeus)* is a small, nocturnal, insectivorous mammal native to Europe. The aim of the study was to describe the arterial supply to the head of the Western European hedgehog in detail and compare it with known vascular patterns found in mammals. In the study, 30 specimens of adult Western European hedgehogs were used. Three different angiological techniques were used to obtain the full image of arterial vascularization of the head in the described species - latex preparation, corrosion cast and contrast-enhanced cone-beam computed tomography. The last of them is a method also used in veterinary practice, which makes the description useful not only to anatomists, but also veterinary clinicians. The most important features of the study are: the species has an interesting, individually specific course of the stapedial artery, in which two different variants have been found. In one of them the vessel provides blood as far as the orbit, but in the other one ends at the temporal region; the auricular region is supplied by branches from the superficial cervical artery; there is no maxillary artery; the occipital artery originates from the internal carotid artery. The results of this work may contribute to establishing new veterinary protocols for the species that is declining in number in many countries and is treated by veterinarians in wildlife rehabilitation centers. It may be also used by clinicians who work with other species of hedgehogs kept as pets.

## Introduction

The Western European Hedgehog *(Erinaceus europaeus)* is a small, terrestrial, insectivorous mammal native to most central European countries. It represents the order Eulipotyphla along with other species and hedgehogs, shrews and moles. It is one of 17 species of hedgehogs living in the world. In 2006, it was assessed for The IUCN Red List of Threatened Species and listed as a species of Least Concern. Western European hedgehogs live natively in forests and grasslands, but the increasing urbanization of European countries forced many hedgehogs to live in urban areas. They can be frequently met in parks and gardens, even in highly developed cities (Amori 2016). They are essential in regulating the invertebrate population they prey on and may be important indicators of human environmental impact (Williams et al. 2018). In recent years, there have been reports of the decline of the Western European hedgehog population have been influenced by various causes (Rasmussen et al. 2024). As the number of hedgehogs in urban areas increases, they are becoming more frequent patients in Wildlife Rehabilitation Centers (WRC). These organizations work in different European countries, treating thousands of hedgehogs yearly (Bearman-Brown and Baker 2022; Kadlecova et al. 2023). In WRCs hedgehogs undergo different basic medical procedures, such as antiparasitic treatment, wound treatment, as well as advanced surgeries (Van de Weyer et al. 2023; Molina-Lopez et al. 2024; Berger 2023). Additionally, the medicine of other species of hedgehogs, especially the four-toed hedgehog *(Atelerix albiventris)* is progressing rapidly, which is a direct consequence of this species being kept as a pet (Okada et al. 2018; Wozniak-Biel et al. 2015). The arterial vascularization of the head of the Western European hedgehog has been partially described in a basic manner. The small group of specimens were used by previous authors, which did not include individually specific variations of the vascular patterns. This study aims to describe the detailed pattern of arteries supplying blood to the head in this species and compare it with other known vascular patterns found in other mammalian species. Hypothesis is: Vascular pattern of arteries supplying blood the head in the Western European hedgehog (*Erinaceus europaeus*) is like one found in other members of the order Eulipotyphla. The results of the study may also help veterinary clinicians establish new medical protocols for hedgehogs, improve surgical techniques, and avoid complications, e.g., hemorrhage. It may be helpful in the future, since its populations are decreasing, and veterinary care may be an important part of the conservation actions aimed to protect the species. The results may also be used in further anatomical studies, including vascular or comparative anatomy on other species belonging to the order Eulipotyphla.

## Materials and methods

### Animals

Therefore, Western European hedgehogs are protected by Polish law (Regulation of the Minister of the Environment of 16 December 2016, on the protection of animal species (Journal of Laws, item 2183) and (Journal of Laws 2020, item 26), all procedures done to accomplish the goal of the study were approved and carried out following the appropriate regulations and permits (Regional Directorate for Environmental Protection in Poznan (Poland): WPN-II.6401.366.2020.TE).

30 analyzed specimens were delivered post-mortem to the Department of Animal Anatomy at Poznan University of Life Sciences from a wildlife rehabilitation center in Poznan, Poland. All animals included in this study had been euthanized with intravenous pentobarbital (Exagon, Richter Pharma AG, Austria, 100 mg/kg) preceded by intramuscular ketamine (Ketamidor, Richter Pharma AG, Austria, 50 mg/kg) and xylazine (Xylapan, Vetoquinol Biovet, Poland, 20 mg/kg) for medical reasons other than for neurological or cardiovascular disease.

The animals were adults, consisting of 15 males and 15 females. The cadavers were frozen in -20 degrees Celsius 15 min after death. Animals with visible trauma to the head or neck region was excluded from the study. The animals had not been euthanized for the purpose of the research.

### Methods

To create angiological specimens, the access to thoracic part of the descending aorta was obtained. To access the vessel, the skin, muscles and fasciae were cut in the linea alba from the sternum to the pelvis. The diaphragm was cut in an arc near the insertions of the costal and sternal parts cut to access the thoracic cavity.

13 randomly selected cadavers were used to create a corrosion cast specimens. The specimens were processed by injecting a COLOREX^®^ (Śnieżka, Poland) red-stained solution of the chemo-setting acrylic material Duracryl^®^ Plus (SpofaDental, Czech Republic) into the descending aorta from the caudal side. After a short time (15–20 min) necessary for setting, the specimens were enzymatically macerated with the detergent (Persil^®^ powder; Henkel, Germany) and diluted in water at 43 °C for 14 days. This procedure resulted in corrosion castings of the vessels on a bone scaffold (without the soft tissues).

The second method, applied to 12 specimens, consisted of passing the liquid-stained latex LBS 3060 (Synthos, Poland) into the thoracic part of the descending aorta from the caudal side, leaving it to set in a 5% formalin solution (Chempur, Poland) for 14 days, then preparing the blood vessels manually using surgical instruments during dissection, to view them within the tissue.

The third method, angio-CT examination, was used in 5 specimens. Prior to the scans, the thoracic part of the descending aorta was injected with barium sulfate (barium sulfuricum 1.0 g/mL, Medana, Sieradz, Poland), as a contrast agent to help visualize arteries in the contrast-enhanced examination. The heads were fixed motionless on the tomograph’s table with the use of adhesive tape. Cone-beam computed tomography (Fidex Animage, Pleasanton, CA, USA) was performed at the University Centre for Veterinary Medicine in Poznan, Poland, with scanning parameters of 110 kVp, 0.08 mAs per shot, 20.48 mAs (Total mAs), and a reconstructed slice thickness of 0.3 mm. After the examination, the scan was post-produced in FidexGUI (version 3.6.0, Animage, USA) to obtain a 3D reconstruction of the arterial vessels. All the excess of tissue, except for bone tissue and arteries was cut out digitally, and the reconstruction was digitally colored in red.

All of the preparations were photographed and described in detail. A digital camera (Canon EOS 250D) with a macro photography lens (IAOWA 100 mm, F2.8) was used to obtain the photographs. The photographs were saved in JPG format. GIMP v2.10.18, digital image editing software, was used to process the photographs.

To better visualize vessels overlapping with bones and clarify different vascular patterns, schemes were drawn in Procreate using 6th generation iPad tablet with Apple Pencil Pro (Apple, USA).

## Results

The blood is delivered to the head mainly by the common carotid artery *(arteria carotis communis)*. It is a bilateral vessel. The left artery branches off from the aortic arch (*arcus aortae*), when the right artery branches off from the brachiocephalic trunk (*truncus brachiocephalicus*). At the level of the second cervical vertebra, this vessel divides into the external carotid artery (*arteria carotis externa*) and the internal carotid artery (*arteria carotis interna*).

**Fig. 1:**
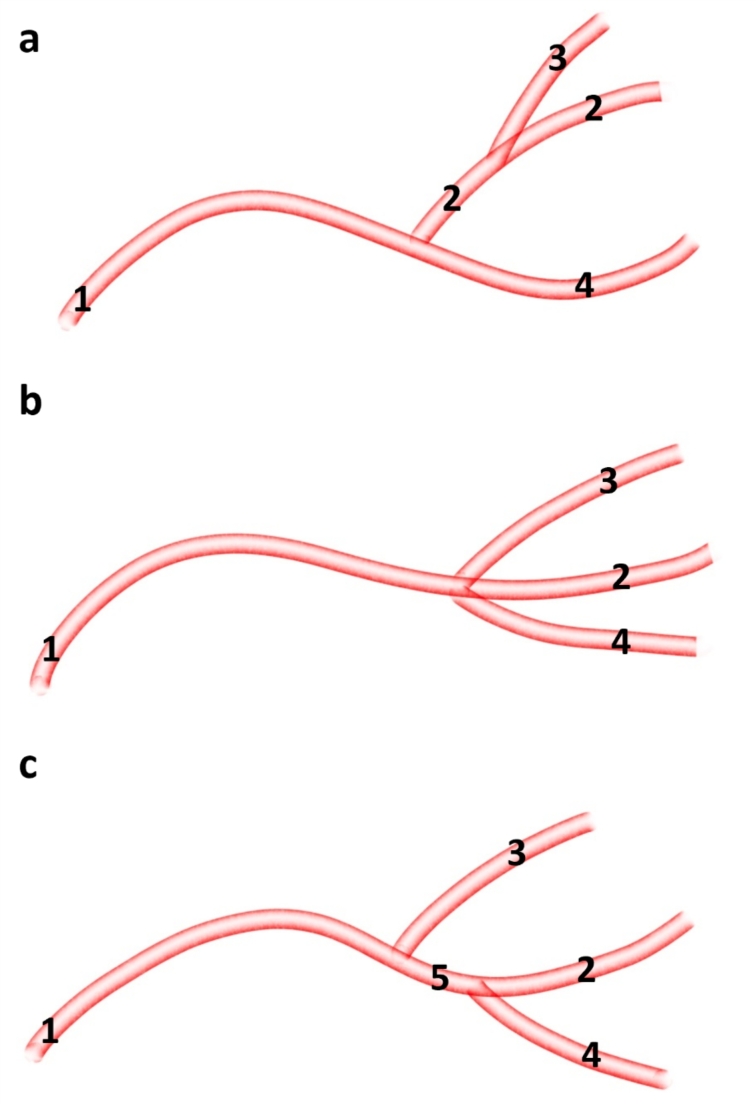
Variants of the external carotid artery branches. 1– external carotid artery 2– facial artery 3– transverse facial artery 4– lingual artery 5– linguo-facial trunk

In most cases (14 out of 30; 47%) from the external carotid artery branches off the facial artery (*arteria facialis*). From this vessel, in its initial segment, the transverse facial artery (*arteria transversa faciei*) branches off (Fig. 1a). After the facial artery branches off, the external carotid artery evolves into the lingual artery (*arteria lingualis*). The lumen of this vessel is like the lumen of the external carotid artery, and the lumen of the facial artery and the transverse facial artery are much smaller. In 13 (43%) of the specimens all three vessels, i.e. the facial artery, the transverse facial artery and the lingual artery branch off in one point (Fig. 1b). In 3 (10%) of the specimens, from the external carotid artery branches off the transverse facial artery. More rostrally the, the facial artery and the lingual artery create the common trunk i.e. the linguofacial trunk *(truncus linguofacialis)* (Fig. 1c). This is a short vessel, which divides into two main components.

**Fig. 2:**
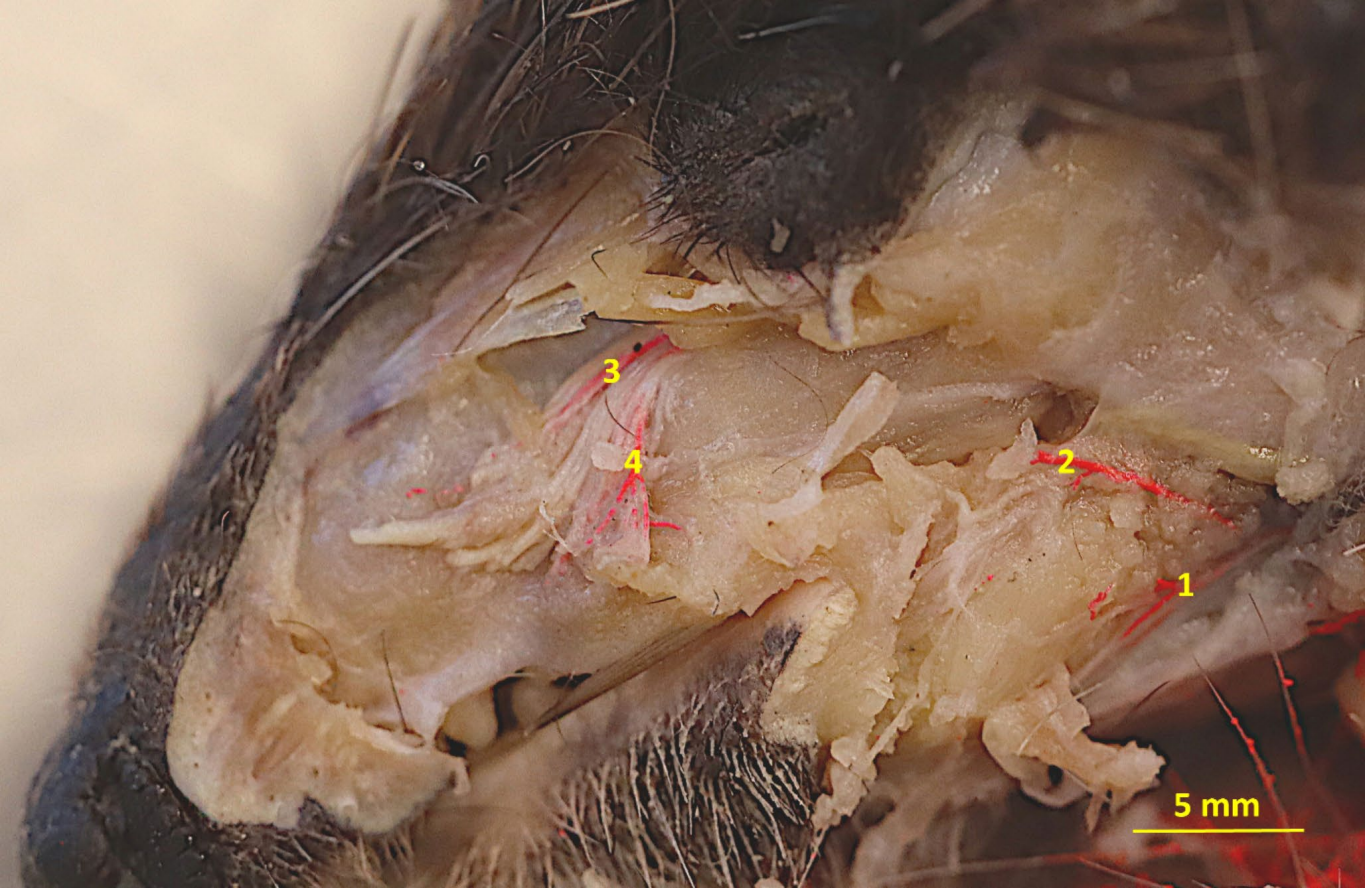
Latex specimen of the superficial arteries of the face region. 1– inferior labial artery; 2 - angular artery of the mouth; 3– nasal branches; 4– superior labial artery

The facial artery wraps around the ventral margin of the mandible in the vascular impression. On the facial surface, the masseteric branch (*ramus massetericus*) branches off, which supplies the ventral part of the masseter muscle. Next, the facial artery divides into an inferior labial artery *(arteria labialis inferior)*, and the angular artery of the mouth *(arteria angularis oris)* (Fig. 2).

The sublingual artery *(arteria sublingualis)* branches off from the lingual artery. This vessel supplies the muscles and skin under the tongue. The lingual artery evolves into the deep lingual artery *(arteria profunda linguae)*, from which the dorsal lingual branches *(rami dorsalis linguae)* branch off. Before the transverse facial artery wraps around the caudal edge of the ramus of the mandible, the glandular branches *(ramus glandularis)* to the parotid and mandibular salivary glands branch off. Next, the transverse facial artery supplies the masseter muscle. This is the main vessel for this muscle. From the initial segment of the transverse facial artery, the rostral auricular artery *(arteria auricularis rostralis)* branches off. It is a weak vessel which supplies the rostral part of auricle muscles.

**Fig. 3:**
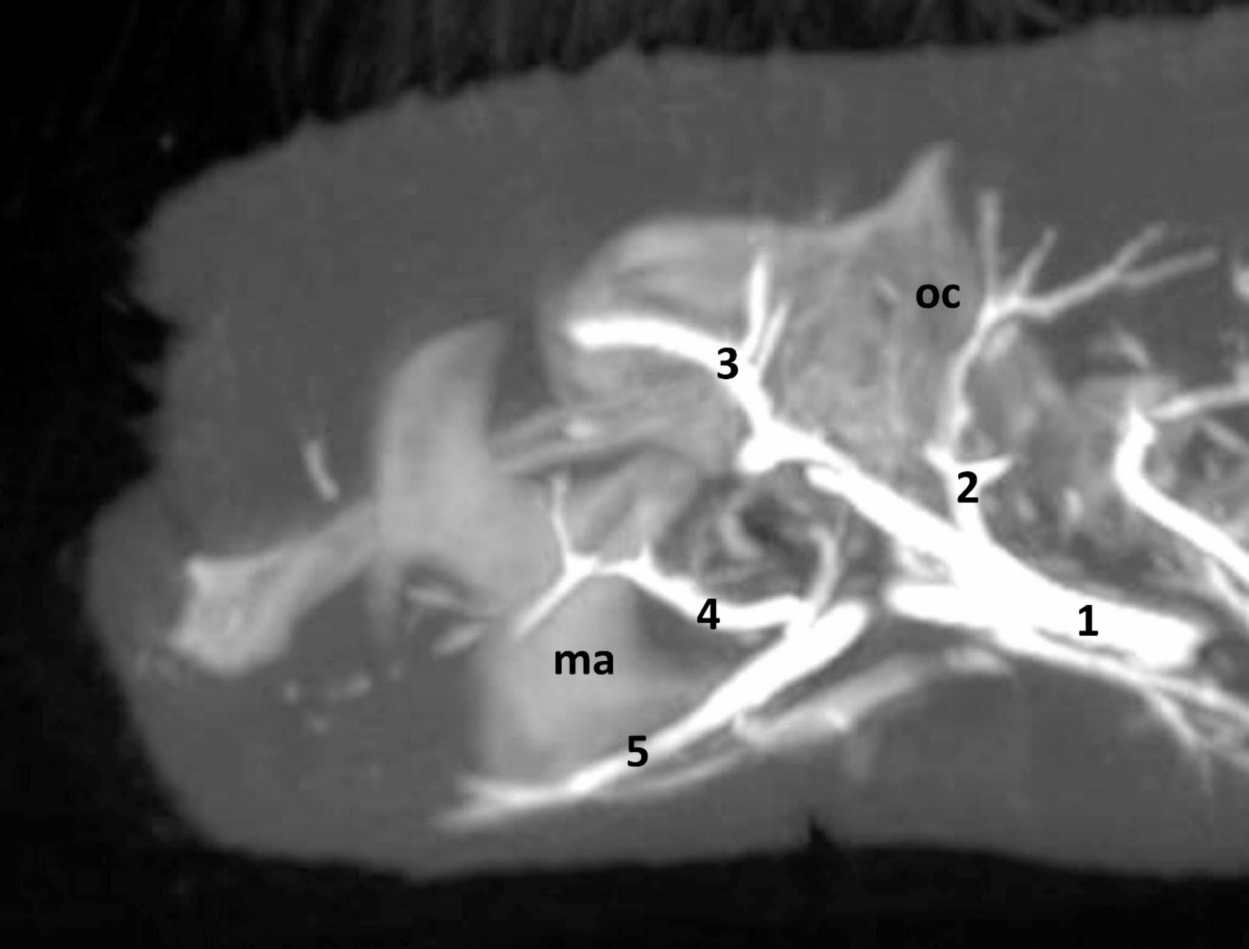
Oblique projection of maximum intensity projection reconstruction of the cone-beam computed tomography scan. Arteries of the caudal part of the head. 1– common carotid artery; 2– occipital artery; 3– superior branch of the stapedial artery; 4– transverse facial artery; 5– facial artery; oc– occipital bone; ma– mandible

From the initial part of the internal carotid artery, the ascending pharyngeal artery *(arteria pharyngea ascendens)* branches off. Moreover, near this point, the occipital artery *(arteria occipitalis)* branches off (Fig. 3).

**Fig. 4:**
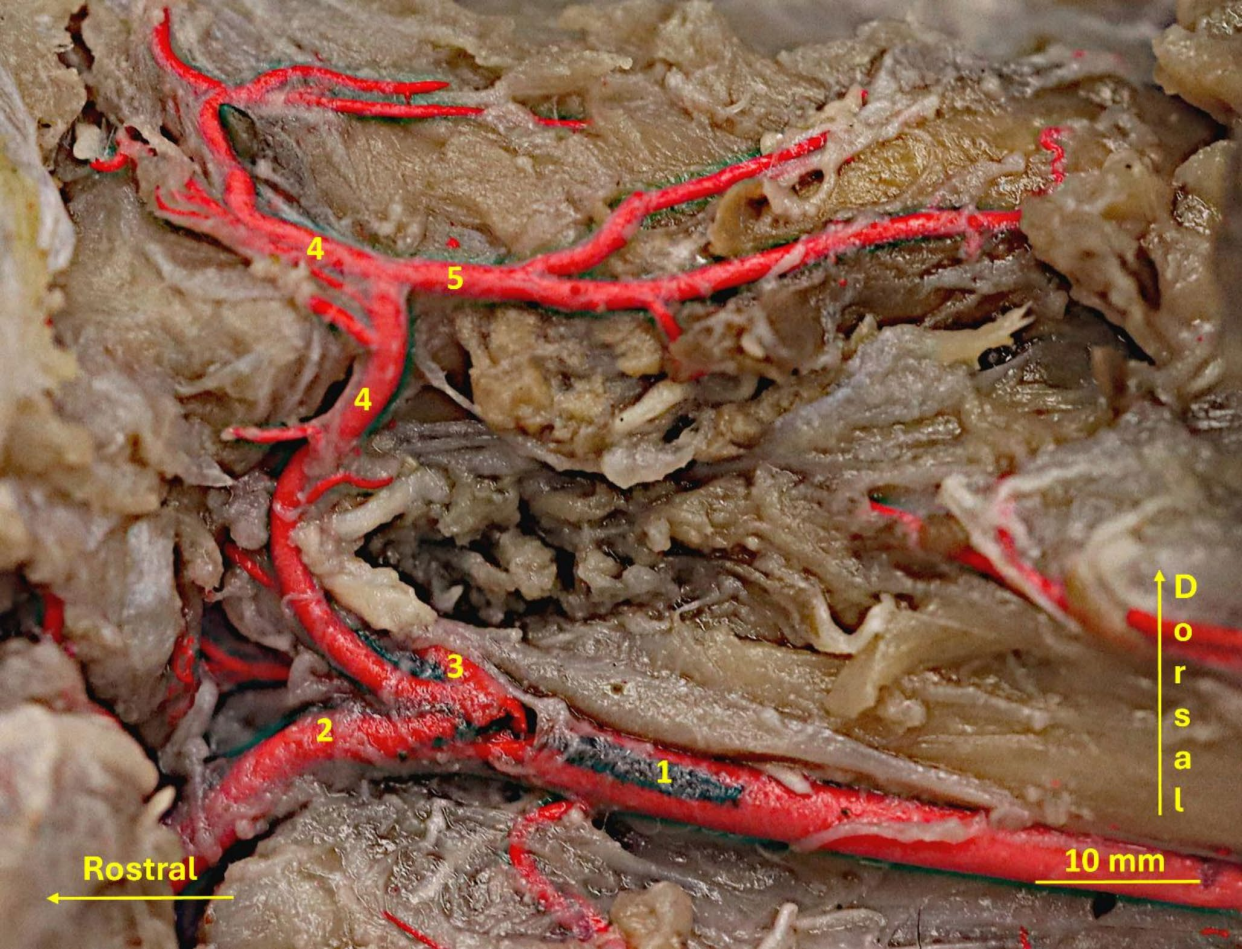
Latex preparation of the arteries of the neck region. 1– common carotid artery; 2– external carotid artery; 3– internal carotid artery; 4– occipital artery; 5– branches to the cervical muscles

This artery heads to the external surface of the occipital bone supplying the muscles of the neck and branching off the small vessel to the horizontal part of the external auditory meatus (Fig. 4).

**Fig. 5:**
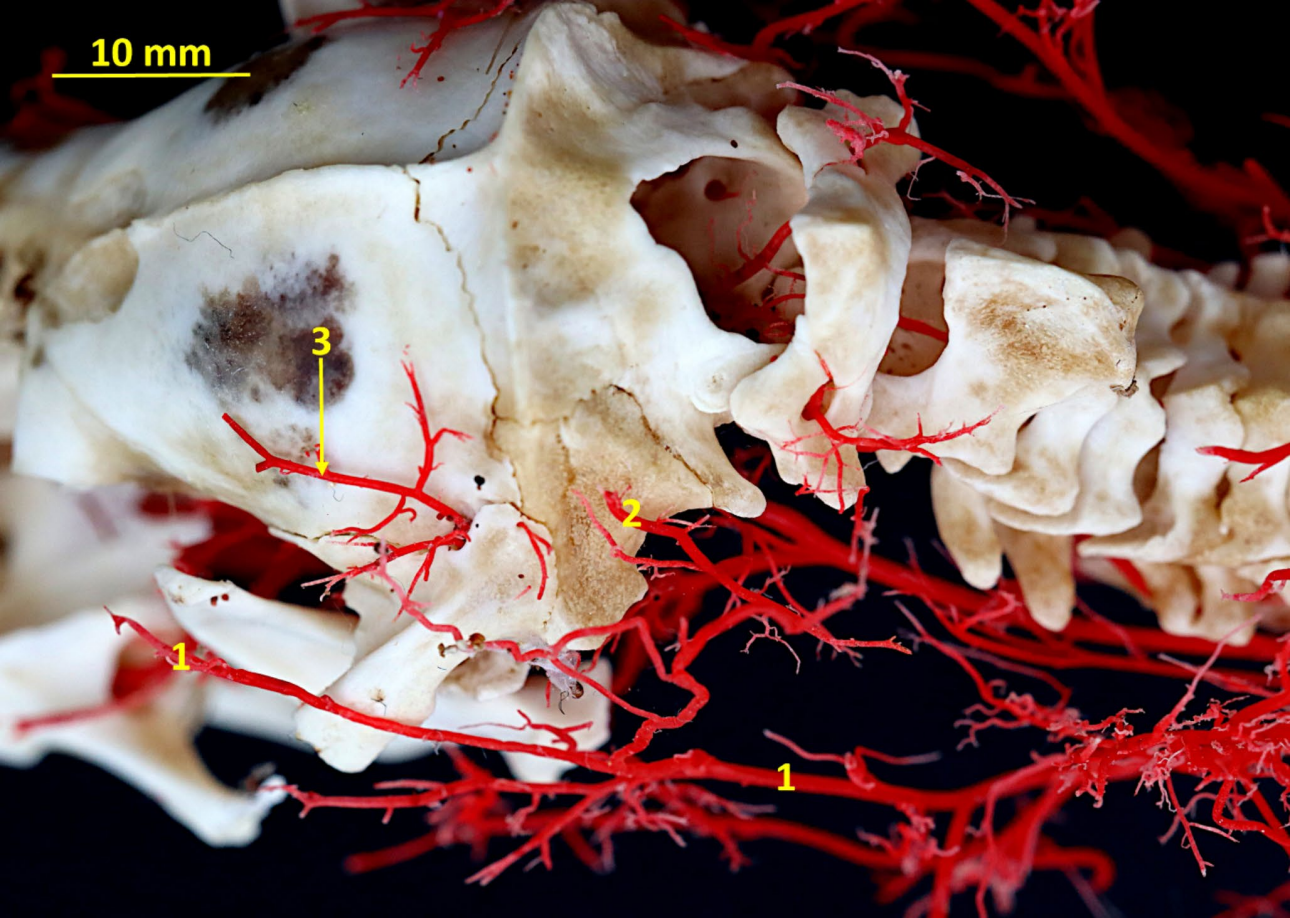
Corrosion cast specimen of arteries of the occipital region. Caudolateral view. 1– superficial cervical artery; 2– occipital artery; 3– temporal branch of the superior branch of the stapedial artery

More superior, the internal carotid artery penetrates the carotid foramen and contributes to cerebral vascularization. Next, the main arterial stream, as the stapedial artery *(arteria stapedia)* passes through the stapes and further forward. It is divided into two rami - the superior and inferior one. From the superior ramus, one small temporal branch (ramus temporalis) branches off and penetrates the temporal meatus and exits through foramina ad meatus temporalis at the external surface of the skull (Fig. 5).

**Fig. 6:**
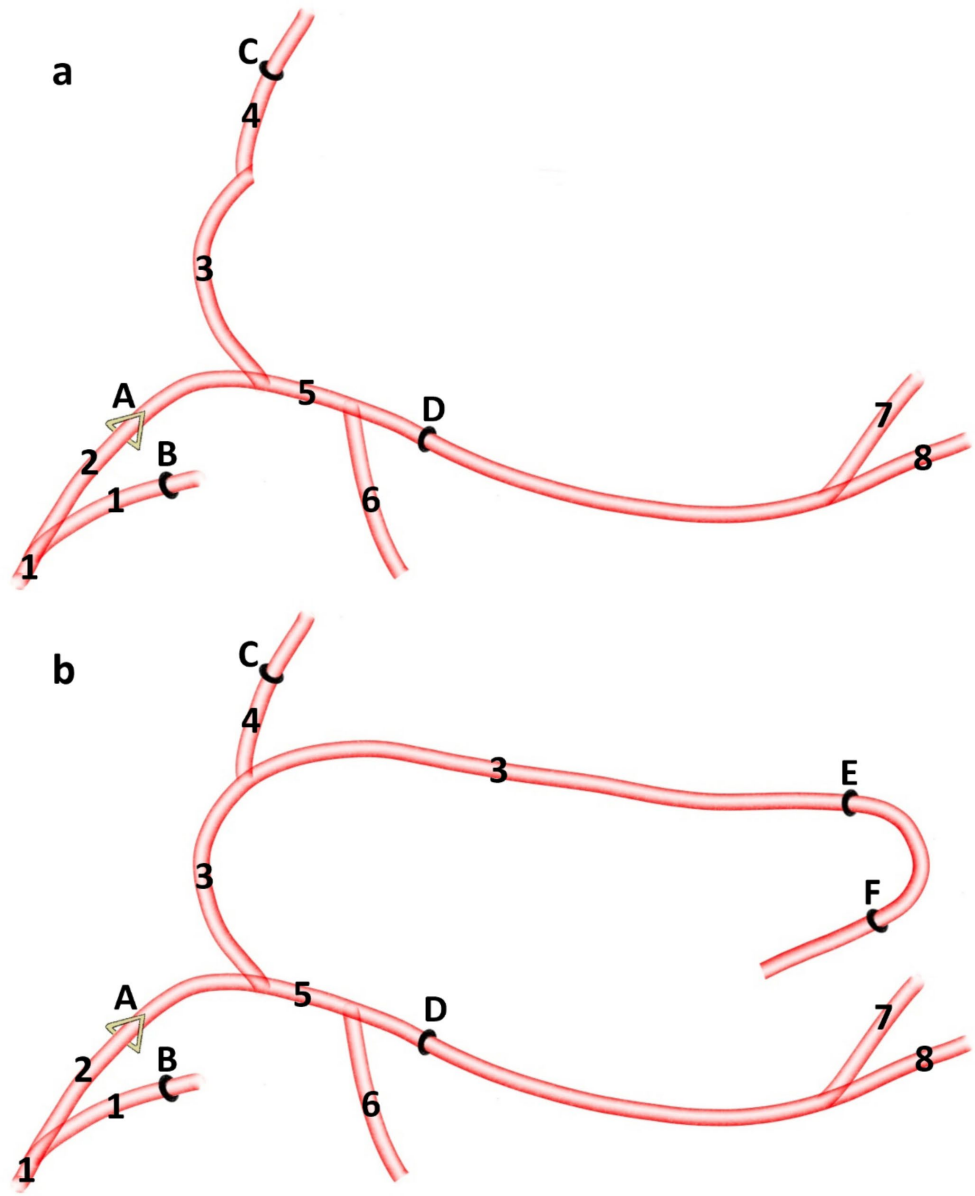
Branches of the stapedial artery. 1– internal carotid artery; 2– stapedial artery; 3– superior branch of the stapedial artery; 4– temporal branch; 5– inferior branch of the stapedial artery; 6– inferior alveolar artery; 7– external ophthalmic artery; 8– infraorbital artery; A– stapes; B– carotid foramen; C - temporal meatus; D– alar canal; E– superior ethmoid foramen; F– inferior ethmoid foramen

This vascular system (variant a) is present in 12 of specimens bilaterally, and in three cases unilaterally, on the right side, and two cases on the left side. In the remaining specimens (variant b) apart from the branch described above, this part of the stapedial artery continues as a strong vessel that lies on the ventral surface of the cranial cavity, runs rostrally, penetrates the superior ethmoid foramen from the internal part of the orbit (Fig. 6).

**Fig. 7:**
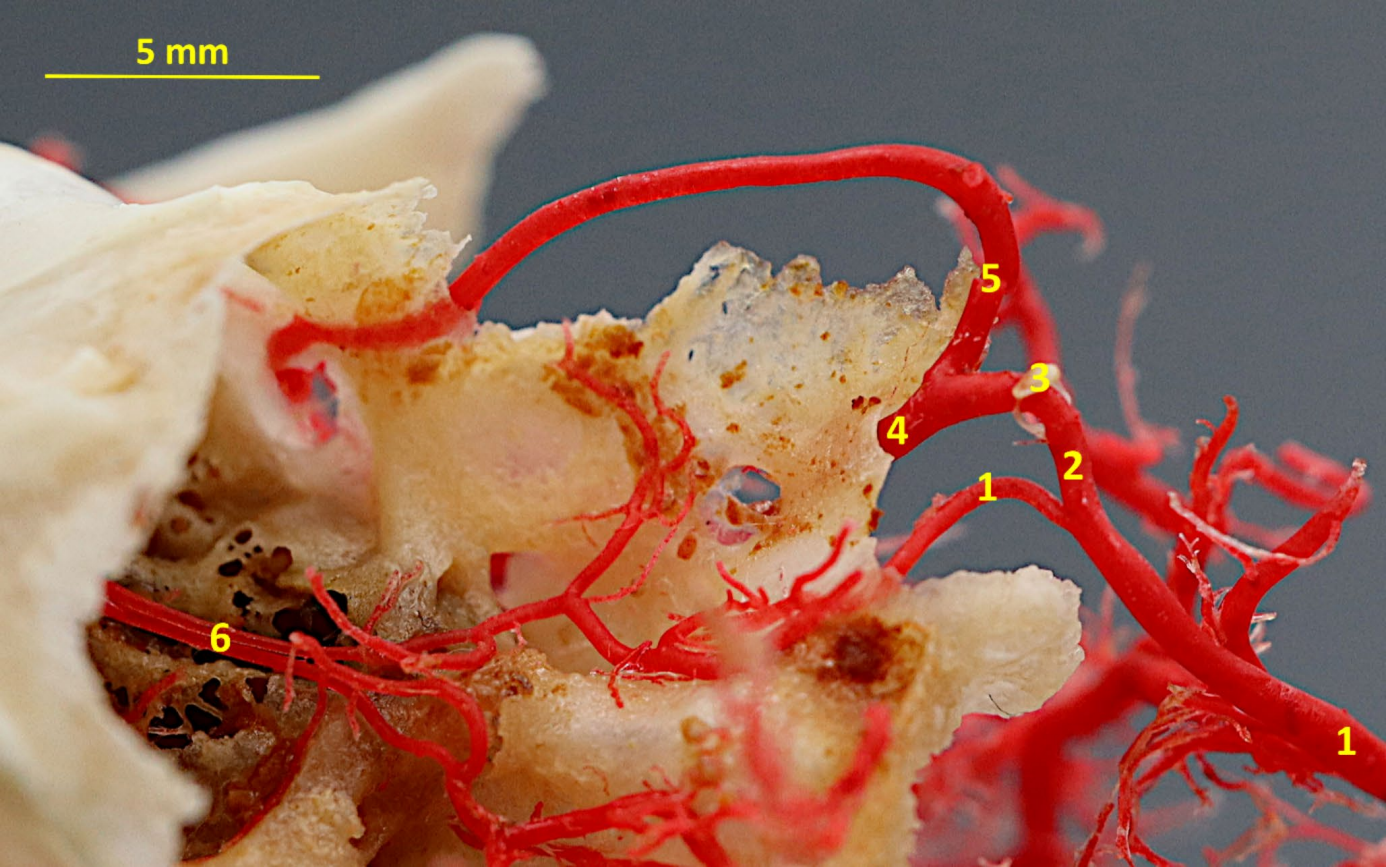
Corrosion cast specimen of the stapedial artery branches. Medial view. 1– internal carotid artery; 2– stapedial artery; 3– stapes; 4– inferior branch of the carotid artery; 5– superior branch of the carotid artery; 6– internal ethmoid artery

Here, a small branch of this vessel creates the supraorbital and frontal arteries (*arteria frontalis*). This vessel wraps around the supraorbital edge, supplying the superior muscles of the skull. Next, the further part of the main vessel enters the inferior ethmoid foramen and forms the external ethmoid artery, which penetrates the ethmoid foramina of the lamina cribrosa of the ethmoid bone (Fig. 7).

In the variant A, above-mentioned vessels branched off from the external ophthalmic artery.

Apart from this vessel, the internal ethmoid artery supplies this area near the crista galli. This vessel branched off from the arterial circle of the brain.

**Fig. 8:**
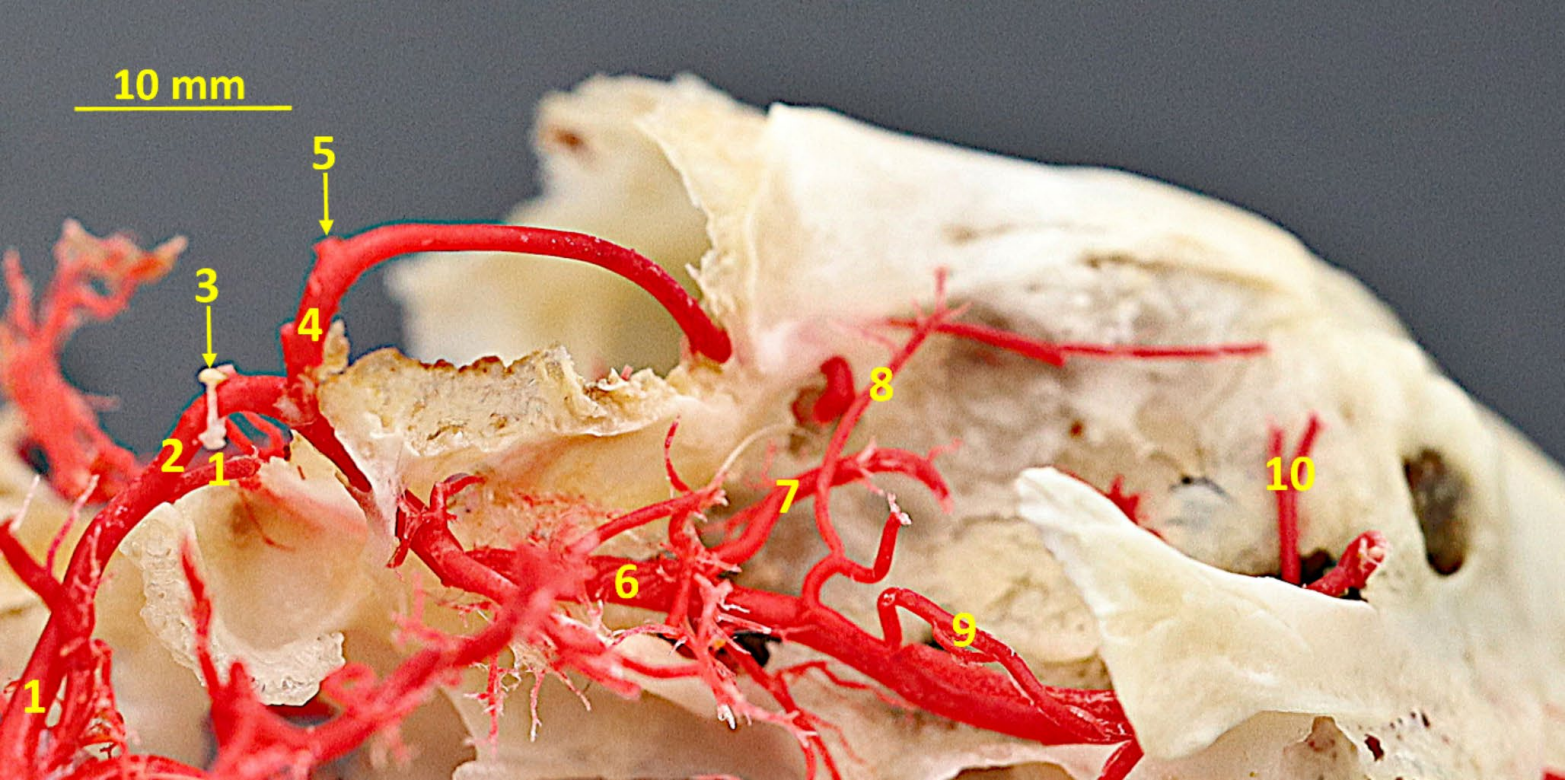
Corrosion cast specimen of the stapedial artery branches. Lateral view. 1– internal carotid artery; 2– stapedial artery; 3– stapes; 4– superficial branch of the stapedial artery; 5– temporal branch; 6– inferior branch of the stapedial artery; 7– external ophthalmic artery; 8– deep caudal temporal artery; 9– buccal artery; 10– malar artery

The second branch, ramus inferior, lies more ventrally and heads rostrally. From it, as the first one, the inferior alveolar artery *(arteria alveolaris inferior)* branches off. From the initial segment of this vessel, the deep caudal temporal *(arteria temporalis profunda caudalis)* artery branches off. From the last-mentioned artery, and from the inferior alveolar artery the pterygoid branches branch off. The inferior alveolar artery penetrates the mandibular canal. Inside the canal, it gives off the dental branches. Finally, this artery leaves this canal by the mental foramen, as mental artery *(arteria mentalis)* (Fig. 8).

More rostrally, the inferior branch of the stapedial artery penetrates the alveolar canal by the caudal alveolar foramen and leaves through the rostral alveolar foramen. Next, the external ophthalmic artery *(arteria ophthalmica externa)* branches off. Its branches penetrate the eyeball and extraocular muscles. Moreover, from this artery, the external ethmoidal artery *(arteria ethmoidalis externa)* branches off. This vessel penetrates the ethmoid foramen. In the cranial cavity, this artery lies on the ethmoid lamina and penetrates the foramina of the ethmoid bone (variant A of the superficial branch of the stapedial artery). Further, the lacrimal artery *(arteria lacrimalis)* branches off. This vessel supplies the lacrimal gland. In some cases, the lacrimal artery branches off from the rostral temporal artery.

**Fig. 9:**
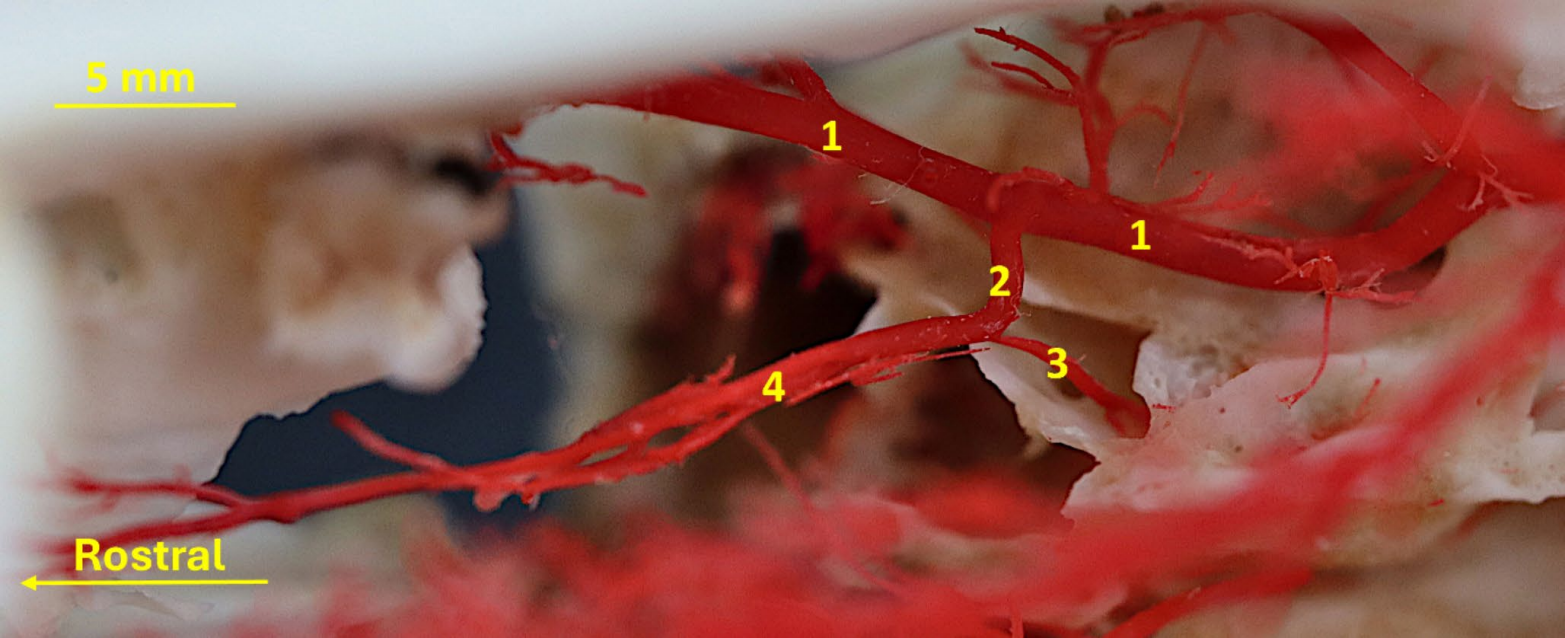
Corrosion cast specimen of the branches of the descending palatal artery. 1– ventral branch of the stapedial artery; 2– descending palatal artery; 3– sphenopalatine artery; 4– major palatal artery

The next vessel branching off from the arterial mainstream is the descending palatine artery *(arteria palatina descendens)* (Fig. 9).

Very close to the point where this artery arises, the small palatine artery *(arteria palatina minor)* branches off. This Is a very weak vessel, which penetrates the small palatine foramina and supplies the soft palate. From the descending palatine artery, the sphenopalatine artery *(arteria sphenopatalina)* branches off. This vessel penetrates the nasal cavity from its caudal part. Next, the descending palatine artery evolves into the greater palatine artery *(arteria palatina major)*. This artery exits the greater palatine foramen and supplies the hard palate.

**Fig. 10:**
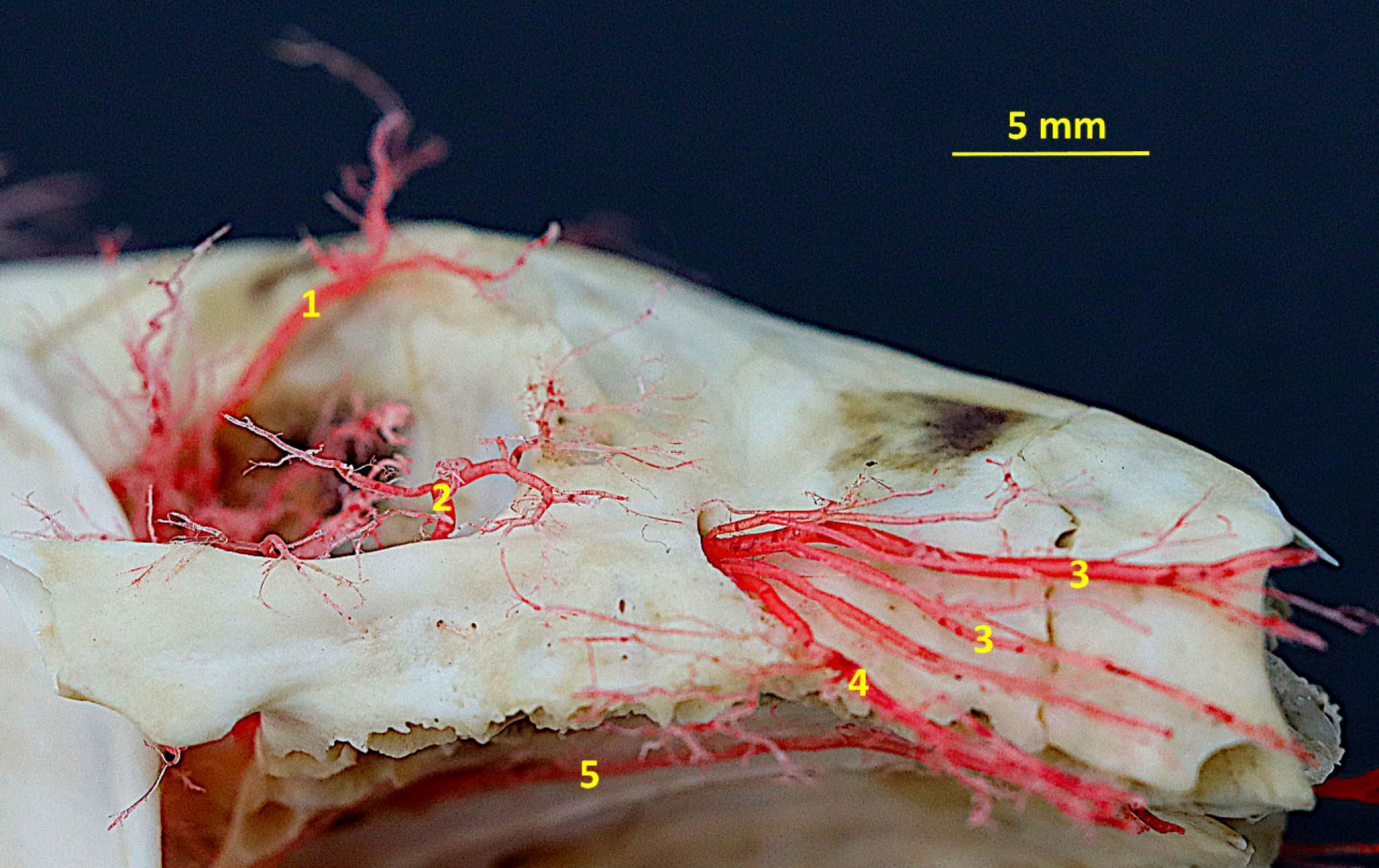
**A**rteries of the face. Corrosion cast specimen. 1– supraorbital artery; 2 - malar artery; 3– nasal branches; 4– superior labial artery; 5– major palatal artery

More rostrally, the deep rostral temporal artery (*arteria temporalis profunda rostralis*) branched off. This vessel supplies the temporal muscle from its rostral side. From this artery, the buccal artery branches off, which supplies the muscles and skin of the cheek. In 10 of specimens this artery branches off from the arterial mainstream, i.e. from the inferior branch of the stapedial artery. Next, the malar artery *(arteria malaris)* branches off. It supplies the region of the medial angle of the eye. Next, the arterial mainstream runs further as the infraorbital artery *(arteria infraorbitalis)*. It penetrates the maxillary foramen. In the infraorbital canal, this vessel gives off the dental branches. After the exit of the infraorbital foramen, the infraorbital artery divides into 2–3 lateral nasal branches *(rami lateralis nasi)* and the superior labial artery (*arteria labialis superior)* (Fig. 10).

The next vessel that participates in head vascularization is the superficial cervical artery *(arteria cervicalis superficialis)*. This vessel branches off from the subclavian artery *(arteria subclavia)*. This artery supplies the muscles of the neck, muscles involved in rolling up, and the region before the shoulder joint. Moreover, this vessel creates the caudal auricular artery *(arteria auricularis caudalis)*, which divides into several, from 2 to 4 vessels, that supply the auricle.

## Discussion

Studies conducted on the vascularization of the head in different orders of animals show great variation in the pattern of blood supply to this part of the body. While the terminal branches usually take their name from the organ they supply, considerable differences can be observed in the course, as well as in the naming, of the vessels that supply blood to these terminal branches.

In dogs, horses, and Pen-tailed tree shrew, the member of the order Scadentia, listed before as Insectivora, the occipital artery branches off from the external carotid artery. In pigs and cows, it branches off from the common carotid artery (Nickel and Schwarz 1963; Wible and Zeller 1994). In adult cats, it branches off from a carotid sinus, but in fetuses and juvenile cats from a common carotid artery or external carotid artery (Ziemak et al. 2021). In the rock hyrax this vessel branched off from the common carotid, external carotid or internal carotid artery (Lindahl and Lundberg 1946). The pattern of vascularization of this area, most like this described in Western European hedgehog, where it branches off from the initial part of the internal carotid artery, was described in a red squirrel, where the occipital artery forms a common trunk with an internal carotid artery (Zdun et al. 2023).

An important role in the vascularization of a large area of the head in the analyzed species plays the stapedial artery. The stapedial artery is also referred to as the orbital, temporal, or orbitotemporal artery, according to some authors (Wible 1987). In most embryos of mammals, this artery provides blood to the supraorbital, infraorbital and mandibular regions. In many adults also such observations have been made (Wible 1984). Our observations are confirmed by the above statement. Wible (1987) noted that in some rodents, insectivores and Microchiroptera the ramus posterior of the stapedial artery supplies the back of the tympanic cavity and the mastoid region. We did not observe this vessel in a Western European hedgehog. This vessel is also not mentioned by Bugge in any of his works on this vessel in numerous rodents (Bugge 1970, 1971a, b, c). Although the topic of major head vessels in the hedgehog was developed by Bugge (1972, 1974), our study showed differences from the information in his studies. In our study, we showed some variants of vessel departure, as well as individual variations. The lack of this information in Bugge’s work may be due to the small number of specimens analyzed, as he only compiled 6 specimens. In some specimens used in our study we did observe variant A, in which the superior branch of the stapedial artery was significantly reduced and created only the temporal branch. Bugge did not mention other variants of this vascular pattern in his works (1972, 1974). Even though these articles do not pay special attention to the vessels branching off from the external carotid artery, in our study we showed 3 variants of the branching off of the arteries: lingual, facial, and transverse facial. The diagram showing this area in Bugge shows the independent departure of the lingual and facial arteries, as well as the transverse facial, which branches off much further from the earlier mentioned ones. Our study showed that those vessels branch off close to each other, and in one variant even showed that the transverse facial artery diverges from the facial artery.

The linguofacial trunk has been observed before in rats, squirrels, Equidae, and most of ruminants except for giraffe and reindeer (Popesko et al. 2010; Kowalczyk and Frąckowiak 2017; Zdun et al. 2014, 2023). The trunk was absent in tapirs, rinos, hippos, some Suiformes, Pen-tailed tree shrew, and some rodent species e.g. kangaroo rats and spiny pocket mouse. (Brylski 1990; Kowalczyk and Frąckowiak 2017, 2019; Wible and Zeller 1994). The different pattern of arteries in the described area was described in camels and llamas, where the facial artery branches off together with the caudal auricular artery (Kowalczyk et al. 2018).

In many described species, the superior labial artery branched off from a facial artery (Nickel and Schwarz 1963; Zdun et al. 2014, 2023). Among the exceptions from this pattern are goat, sheep, springbok, saiga, Barbary sheep, mouflon where this vessel branches off from the transverse facial artery (Nickel and Schwarz 1963; Zdun et al. 2014). Another pattern, as described in this study, where this vessel branched off from the infraorbital artery, has been described before in giraffe, Roe deer, reindeer, rhino, tapirs, pig, peccary, and desert warthog (Zdun et al. 2014; Kowalczyk and Frąckowiak 2017, 2019).

The facial artery was absent in some ruminant species e.g. sheep, goat, mouflon, saiga (Zdun et al. 2014).

The caudal auricular artery branched off from an external carotid artery in dogs, cats, goat, sheep, cow, pig, and horse (Nickel and Schwarz 1963). In camels and llamas, it branched off the common trunk with the facial artery (Kowalczyk et al. 2018). In Western European hedgehogs, this vessel branched off from a well-developed superficial cervical artery. This pattern is unique for the described species and has not been described before in other species.

The next difference involves the vascular system within the orbit. Bugge (1972, 1974) indicates that obliteration of the internal ophthalmic artery occurs. This is confirmed in our study, as we found no occurrence of this vessel in any of the individuals analyzed. He claims that an a1 anastomosis is formed, connecting the lacrimal artery and the vessels supplying the eyeball. These vessels supplying the eyeball, in both our and Bugge’s, diverge from the a2 anastomosis branching off from the inferior branch of the stapedial artery. In our study, the a2 anastomosis was named the external ophthalmic artery. However, an a1 anastomosis occurs only in some individuals in which a lacrimal artery branched off from the external ophthalmic artery. In this study, it was shown that in some cases this vessel is branching from the rostral temporal artery. A certain peculiarity of the blood vessels of the head of this species is the absence of the maxillary artery. The maxillary artery is considered by Bugge 1970, 1971a, b, c, 1972), 1974); Wible (1987) as a connection of the external carotid artery and the stapedial artery, precisely the inferior branch of the stapedial artery or one of its final branches, the infraorbital or mandibular one. In this study, we did not find such a connection. The absence of a maxillary artery is also characteristic of the chrysochlorids e.g. *Chrysochloris asiatica* and *Amblysomus hottentotus*; certain tenrecids e.g. *Echinops telfairi*; soricids e.g. *Sorex araneus*, *Neomys fodiens* and *Suncus murinus tytleri* and certain erinaceids e.g. *Hemiechinus auritus* (Bugge 1974) dipodoids (Bugge 1972), hamsters and certains New World mice e.g. Onychomys and Peromyscus; voles and Apodemus (Bugge 1970).

The anatomical system of arteries supplying blood to the head, despite the existence of several variants, has a very interesting feature. The course of the external carotid artery and its branches determines the fact that, unlike many other mammalian species, this vessel does not supply blood to the eye socket, mandible, maxillary and nose area (Kowalczyk et al. 2018; Kowalczyk and Frąckowiak 2017, 2019; Nickel and Schwarz 1963). The supply range of this vessel in Western European hedgehog is limited to the masseter muscle, lower lip, salivary glands, and part of the tongue. Therefore, damage to this vessel during trauma or iatrogenic, e.g. during surgical intervention, carries the risk of ischemia in a much smaller area of the head. Moreover, the tissues involved in this process are much less important for the animal in terms of a successful prognosis and recovery.

## Data Availability

The data used to support the findings of this study are available from the corresponding author upon reasonable request.
